# Variability as a marker of motor imagery in cerebral palsy: Between intention and action

**DOI:** 10.1111/dmcn.70224

**Published:** 2026-02-28

**Authors:** Deisiane Oliveira Souto

**Affiliations:** ^1^ Graduate Program in Rehabilitation Sciences, Physical Therapy Department, School of Physical Education, Physical Therapy and Occupational Therapy Universidade Federal de Minas Gerais Belo Horizonte Brazil

## Abstract

**This commentary is on the original article by Breuer Asher et al. on pages** 1570–1579 **of this issue.**

Even in the presence of significant motor limitations, how does physical action continue to be intensely elaborated by the brain? Cerebral palsy (CP) requires motor control to be understood not only as peripheral execution but as an internal construction of action. Between the intention to move and the possible action lies a representational domain in which movement is anticipated, organized, and mentally rehearsed, and it is at this conceptual level that motor imagery becomes central.^1^ Disregarding this dimension implies restricting rehabilitation to the observable body while neglecting neural processes responsible for intention and motor planning. Thus, shifting the focus to motor imagery means investigating not only whether the child moves, but how actions are planned and simulated before execution.

This perspective poses a methodological challenge: how to empirically operationalize internal motor simulation processes. The Hand Laterality Test is widely used as an indirect measure of motor imagery because it requires mental rotation of one's own limb for spatial judgment. However, results based only on mean reaction time or accuracy have been inconsistent. In this context, the study by Breuer Asher et al.^2^ is particularly relevant for proposing performance variability as a primary indicator of imagery stability. This proposal aligns with contemporary models of motor control and cognition in CP, in which variability is interpreted as a marker of system instability. Accordingly, performance fluctuations should not be regarded as statistical noise but as manifestations of alterations in internal mechanisms of action simulation, supporting the hypothesis of Steenbergen et al.^3^ that imagery and planning deficits are qualitative and compromise representational consistency.

The study by Breuer Asher et al.^2^ advances the field by showing that motor imagery in children with CP is characterized less by reduced accuracy and more by increased intraindividual variability, particularly in reaction time. By adopting variability as a core variable, the authors provide an integrative explanation for previously discrepant findings, suggesting that the main difficulty lies in performance instability rather than slowness.^3^, ^4^ This interpretation is consistent with a recent systematic review demonstrating substantial methodological and outcome heterogeneity, indicating that imagery deficits vary according to task demands, clinical subtype, and functional level.^5^ Moreover, the proposed composite score integrating accuracy and variability advances the clinical operationalization of motor imagery by offering a quantitative criterion capable of discriminating children with CP from typically developing peers and monitoring this ability over time.

Despite these advances, controversies remain regarding the nature of the motor imagery deficit in CP. It is unclear whether increased variability reflects alterations in specific motor simulation mechanisms or broader limitations related to attention, executive functions, or working memory. Steenbergen et al.^3^ suggested that imagery impairments may be linked to general difficulties in action planning and representation rather than exclusively to simulation capacity. The high prevalence of attention‐deficit/hyperactivity disorder in the sample studied by Breuer Asher et al.^2^ reinforces this complexity, as reaction time instability may reflect attentional fluctuations rather than failures of body representation alone. Thus, it remains unresolved whether variability constitutes a specific marker of imagery or a general index of cognitive instability.

Clinically, these findings indicate that motor imagery should not be applied uniformly to children with CP, given distinct imagery performance profiles. Prior assessment is therefore necessary to guide eligibility and intervention design, as children with greater variability tend to respond less consistently to imagery‐based protocols and may benefit from combined strategies incorporating attentional support and perceptual feedback, consistent with evidence of partial imagery preservation in unilateral CP.^4^ For future research, it will be important to determine whether performance variability is sensitive to training and whether it represents a stable trait or a modifiable state.

## Data Availability

Not required.
